# Death talk: gender differences in talking about one’s own impending death

**DOI:** 10.1186/1472-684X-13-8

**Published:** 2014-03-11

**Authors:** Bragi Skulason, Arna Hauksdottir, Kozma Ahcic, Asgeir R Helgason

**Affiliations:** 1 University of Iceland, Saemundargata 2, Reykjavik IS101, Iceland; 2 National University Hospital, Eiriksgata 29, Reykjavik IS101, Iceland; 3 Centre of Public Health Sciences, University of Iceland, Saemundargata 2, Reykjavik IS101, Iceland; 4 Division of Clinical Cancer Epidemiology, Department of Oncology, Institute of Clinical Sciences, Sahlgrenska Academy, University of Gothenburg, Vasagatan 33, 411 37 Gothenburg, Sweden; 5 Sahlgrenska University Hospital SE, Per dubbsgatan 16, 413 45 Gothenburg, Sweden; 6 Karolinska Institute, Departments of Public Health Sciences and Oncology-Pathology, & Centre for Epidemiology and Community Medicine, Stockholm County Council Health Services, Stockholm SE171 77, Sweden; 7 Reykjavík University, Menntavegur 1, Reykjavik IS101, Iceland

**Keywords:** Death, Terminal care, Communication, Evocation, Gender

## Abstract

**Background:**

According to common practice based on a generally agreed interpretation of Icelandic law on the rights of patients, health care professionals cannot discuss prognosis and treatment with a patient’s family without that patient’s consent. This limitation poses ethical problems, because research has shown that, in the absence of insight and communication regarding a patient’s impending death, patient’s significant others may subsequently experience long-term psychological distress. It is also reportedly important for most dying patients to know that health care personnel are comfortable with talking about death and dying. There is only very limited information concerning gender differences regarding death talk in terminal care patients.

**Methods:**

This is a retrospective analysis of detailed prospective “field notes” from chaplain interviews of all patients aged 30–75 years receiving palliative care and/or with DNR (do not resuscitate) written on their charts who requested an interview with a hospital chaplain during a period of 3 years. After all study patients had died, these notes were analyzed to assess the prevalence of patient-initiated discussions regarding their own impending death and whether non-provocative evocation-type interventions had facilitated such communication.

**Results:**

During the 3-year study period, 195 interviews (114 men, 81 women) were conducted. According to the field notes, 80% of women and 30% of men initiated death talk within the planned 30-minute interviews. After evoking interventions, 59% (67/114) of men and 91% (74/81) of women engaged in death talk. Even with these interventions, at the end of the first interview gender differences were still statistically significant (p = 0.001). By the end of the second interview gender difference was less, but still statistically significant (p = 0.001).

**Conclusions:**

Gender differences in terminal care communication may be radically reduced by using simple evocation methods that are relatively unpretentious, but require considerable clinical training.

Men in terminal care are more reluctant than women to enter into discussion regarding their own impending death in clinical settings. Intervention based on non-provocative evocation methods may increase death talk in both genders, the relative increase being higher for men.

## Background

Health care professionals all over the world are faced with terminal patients every day, and are managing each patient as best they can.

It is currently widely recommended that discussions about end-of-life care begin early in the terminal care process, especially in patients with cancer [1-7]. Discussions between patient and physician about end-of-life care preferences are associated with less aggressive care near death [8-11]. Studies have shown that many physicians avoid end-of-life discussions until death is imminent [12-15].

Every day, health care professionals worldwide face difficult discussions with terminal patients and do their best to conduct them in the light of their clinical training and clinical and personal experience.

Early practices frequently withheld information from terminal patients. In his groundbreaking 1963 survey in Britain, the anthropologist Geoffrey Gorer found that dying people were kept in ignorance about their imminent death, whereas close family members were informed [16]. However, since the 1960s there has been a shift in Western medical ethics, often towards conditional disclosure, not stating worst possible scenario, as opposed to full disclosure of prognosis [17]. Conditional disclosure may be described as a trade-off dilemma between the patients and relatives right to be informed versus the right to hold on to hope, or not to know.

Cicely Saunders, a key person in the establishment of the international Hospice/Palliative Care movement [18], defines active communication as “being with” a person (facing imminent death in a Palliative Care setting). This means not only taking care of the patient’s physical needs, but also being available when the patient wants to discuss spiritual and emotional concerns. The “*person as a whole*” is at the core of Palliative Care [19-30]. Modern day health service providers struggle to meet the ideal of “being with” someone who is dying and providing appropriate care and communication as they face their last days of life. However, not all patients want to talk about emotionally taxing feelings or pain. Also, possible gender differences need to be considered. Martin and Doka have criticized dominant counseling paradigms for privileging the expression of emotion [31]. Their claim is that masculine patterns of grief are different, but no less effective. They talk about “intuitive” and “instrumental patterns” of grief, which are correlated with gender, but not determined by it. According to Martin and Doka’s findings, neither strategy is superior [31]. Studies have demonstrated that men in general and male cancer patients in particular confide only in their partners [32]. However, studies indicate that it is important for most dying patients to know that health care personnel are comfortable with talking about death and dying and that they are welcome to discuss personal fears and worries regarding their own impending death with them [33-35]. Studies of patients for whom cure is no longer possible have shown that few such patients experience discussions about death and dying as stressful; some experience them as helpful [36-38].

Providing family members with pragmatic information requires well-established communication to be in place. It is difficult to operationalize well-functioning communication for everybody involved, but we define well-functioning and established communication as communication that is based on trust and respect for personal boundaries.

Because of generally accepted interpretations of laws protecting patients’ integrity, in many countries, including Iceland, communication between health care professionals and dying patients may be a prerequisite for communication with those patients’ significant others. The issue of providing terminal patients with opportunities to talk about their own impending deaths for the purpose of facilitating communication between health care personnel and those patients’ significant others has several important ethical aspects. That patients have the right to keep such information from relatives does not necessarily mean that they do not want to discuss this subject. Both medical staff and family members are put in difficult situations when patients want total silence about their impending death. Research has shown that the long-term benefits of receiving information about a loved one’s impending death include making it possible to prepare for that death, thus significantly decreasing psychological trauma and morbidity during bereavement [33-35,38-43]. However, this benefit must be weighed against the possibility that some patients wish to avoid talking about their own impending deaths as a way of coping.

Gender has frequently been examined in connection with bereavement. Studies indicate that widowed men have greater mortality rates than non-widowed men for up to 9 years after the deaths of their spouses [44]. Widowed men may be more negatively affected by bereavement than widowed women [45-49]. Some studies have even found widowhood to be a protective health factor for women [50,51], and becoming a widow to be positive for some women’s personal growth [50,52,53]. The roots of such gender differences have been associated with amount and quality of emotional or social support [32,48,54,55] and ability to cope with stress [45,47]. Others have found no association between such gender differences and social support [56]. It has also seen suggested that the gender difference in mortality risks in relation to bereavement may, at least partially, be due to differential health effects of marriage, in that women are more proactive than men about maintaining their health [57].

Widowers’ preparedness before their wives’ deaths from cancer and how it affected their risk of long-term morbidity has been studied. Lower preparedness was shown to increase emotional numbness, grief resolution, and sleep disorders 4–5 years after the deaths of the participants’ wives [41]. Wives’ awareness time concerning their husbands’ impending deaths from cancer has been shown to be influenced by information and psychological support from caregivers [35].

Studies indicate that men may need a gender specific therapeutic approach when seeking support. When men seek help their effort is often hesitant and complicated by conflicting motives, making it difficult for counselors to establish therapeutic alliances [58].

Men have seem to find it difficult to admit that there is a problem, to ask for help, to identify and process emotional states, and to deal with intimacy [59]. Therapists may, therefore, need to develop gender specific forms of psycho-social support to address men’s emotional needs [60-62].

The need to explore gender specific communication style for expressing emotion has been explored in several studies [63-65]. The results suggest that men’s expression of emotion may have been misinterpreted using women’s emotions as gold standard, thus sometimes interpreting men’s emotional signals as absence of emotion [66,67].

The aims of the present study were: a) to assess if dying patients initiate talk about their own impending death with a hospital chaplain; b) to assess gender differences; c) to assess the effect of evocation-type intervention.

## Methods

### Study subjects

The study group comprised all patients aged 30–75 years registered at the National University Hospital in Reykjavík, Iceland from 2006–2008 who were receiving Palliative Care and/or had DNR (do not resuscitate) written on their charts and requested pastoral care. All subjects were attended by the first author (BS). The first author works primarily in the cancer unit where most of the interviews were conducted (Table 1). All subjects were deceased by time of data analysis.

**Table 1:** General characteristics of 195 interviewed dying participants; 175 from terminal cancer care (TCC) and 20 from other terminal care (OTC)

	**All**		**TCC**		**OTC**	
	**Female**	**Male**	**Female**	**Male**	**Female**	**Male**
	**n = 81**	**n = 114**	**n = 71**	**n = 104**	**n = 10**	**n = 10**
30-49 years-old	15%	18%	13%	14%	30%	50%
	(12/81)	(20/114)	(9/71)	(15/104)	(3/10)	(5/10)
50-69 years-old	35%	35%	34%	36%	40%	30%
	(28/81)	(40/114)	(24/71)	(37/104)	(4/10)	(3/10)
70+ years-old	51%	47%	54%	50%	30%	20%
	(41/81)	(54/114)	(38/71)	(52/104)	(3/10)	(2/10)

### Main research questions

Q1: Is there a gender difference in being open to the possibility to engage in talk about one’s own impending death?

Q2: Do evocation-type interventions have an effect on men and women in the same way in relation to talk about own impending death?

### Data collection methods

This is a retrospective analysis of detailed, prospective verbatim field notes of interviews conducted by the first author (BS) in accordance with traditional pastoral care reports. Such notes are written immediately after each interview. They are comparable to the patient’s medical records. *Pastoral verbatim* is based on methods originally developed in ethnography–anthropology to register events and processes during fieldwork: these methods aim to minimize the disturbing effects of registration devices like note writing, tape recording and filming. Field notes in pastoral care are detailed accounts of the actual dialogue. Recording of interview information in the form of detailed field notes is state of the art in pastoral care. Field notes have been used in Clinical Pastoral Education since the 1930s [68], and are an integral part of clinical pastoral care programs within the Association for Clinical Pastoral Education [69]. The method corresponds to the “fieldwork” methodology commonly used in anthropology [70]. A hospital chaplain’s writing and analyzing of clinical field notes may be considered equivalent to writing and analyzing patient records: good clinical practice. Only field notes concerning terminal patients were analyzed.

The standard procedure for these hospital chaplain interviews was not to participate in “death talk” unless the patient initiated this. The context of these interviews is discussed in the Discussion Section.

If death talk had not occurred 30 minutes into the clinical interview, an attempt was made to evoke such talk as a natural part of the process. The interview structure analyzed in the present study had developed from the first author’s clinical experience but there is no clinically documented consensus on how to conduct such interviews. Methods derived from Motivational Interviewing were an integral part of the interview structure [71]. Two important concepts in Motivational Interviewing are “reflections” and “open ended questions”.

A *simple reflection* in an interview means that the counselor repeats what the patient says without changing the meaning of the sentence e.g.

Patient: I have a short time left to live.

Chaplain: Your time is limited.

*Complex reflections* differ from simple reflections in that they open up the possibility to develop the direction and content of the interview by slight alterations in the meaning of the statement e.g.

Patient: I worry about my family.

Chaplain: You’re worried about what will happen to your family when you’re no longer able to be there for them.

A complex reflection thus purposefully alters the meaning of the statement made by the patient to invite deeper conversation.

Reflections simple and complex are defined as statements (never questions) uttered by the counselor (in this case hospital chaplain) in response to something the patient says. The therapeutic rationale for using reflections instead of questions is provided in textbooks on Motivational Interviewing [71].

Questions are then divided into open versus closed. An open ended question is constructed in such a way that it is not possible to answer it with single words like yes or no. One example of an open ended question is: What changes can you see taking place in the future? (See subsection reflection vs. intervention below). A corresponding hypothetical close question would be: Are you hopeful for the future?

### Reflection vs. intervention

#### INTERVENTION - alternative 1: *Direct reflection*

Direct reflection is the primary intervention and is a natural part of the professional interview. The patient can for example touch on the topic without going directly to a discussion on own impending death.

*Patient:* “I worry about my family”.

Example of a *direct reflection*: “You’re worried about what will happen to your family when you’re no longer able to be there for them”.

These kind of statements touching the boarders of death talk followed by a direct reflection taking the issue one step further without confronting the patient’s integrity, usually leads to a talk about own impending death. But not always.

#### INTERVENTION – alternative 2: *Delayed -/re-reflection*

The professional interviewer re-reflects what happened earlier in the interview, for example:

“You stated earlier that you’re worried about what will happen to your family when you’re no longer able to be there for them”.

Please observe that the re-reflection at the end of the conversation only takes place if a direct reflection doesn’t lead to talk about own impending death.

If no talk about own impending death occurred as a result of immediate or delayed re-reflection OR if no statements during the interview gave an opportunity for reflection on death, an open-ended question is asked as a last invitation to talk about own impending death.

#### INTERVENTION alternative 3: *Open-ended question*

What changes can you see taking place in your future?

### Confidentiality and ethics

At the time of the interviews, neither the patient nor the chaplain was aware that the content of the field notes would be subject to analysis and scientific study. After all the patients were deceased, it was suggested that the content of the field notes should be analyzed and the results published in a scientific journal. Approval was obtained from the National Bioethics Committee of Iceland (number VSNb200620033/03.7) and the Icelandic Data Protection Authority (number 2012060771). These, the two main national ethical institutions, both approved the study.

The patients’ acceptance (consent) for the interview was deemed to have been active at two stages, the first being their request for the interview and the second participation in it. If patients decided at any point that they did not want to participate in the interviews, they could discontinue them and were not approached further. Interviews were by referral only. Death talk occurred either spontaneously or as a result of evocation-type interventions.

The names of those interviewed were unidentifiable/untraceable in the field notes subject to analysis in the present study and complied with the same regulations concerning confidentiality and anonymity as do all other hospital patient records. As a rule, all research material derived from field notes or other patient records will be destroyed 5 years after publication, as required by the Icelandic National Bioethics Committee.

Ethical aspects are discussed further in the Discussion Section of this paper under the sub heading “ethical dilemma”.

### Intervention protocol

The content of Hospital Chaplain interviews was not pre-determined. Discussions of the patient’s own impending death were a common theme, but not a necessary part of the clinical interview. The patients controlled the direction of the interviews. In some cases patients initiated talk about their own impending death and more rarely, requested interviews to discuss their own impending death. In these cases all such interviews were registered in the field notes as “patients having initiated death talk”.

If, 30 minutes into the interview, death talk had not occurred spontaneously, or as a result of direct or “delayed reflections” or “delayed re-reflections”, the hospital chaplain attempted to evoke such talk by using an open-ended question (See reflection vs. intervention page 6).

We use the term “delayed reflection” as opposed to “direct reflection” to distinguish between reflections that are uttered by the counselor in direct connection to something stated by the patient. A delayed complex reflection may be uttered as one or more sentences remote from the patient’s statement. If the delayed reflection dose not result in the target behavior (in this case death talk) the same or a similar complex reflection, related to the patients previous statement, may be repeated again later in the interview. To distinguish from delayed reflection, we refer to such utterances by the counselor as “delayed re-reflections” (See reflection vs. intervention page 6).

If the patient did not respond to the interventions the interviews were concluded; however, if they did respond the interviews continued. If a patient asked for an additional interview it was granted. It’s important to keep in mind that these are terminal patients who have been informed by their doctor that they’re suffering from an incurable disease, and many of them have DNR written in their chart. As a result of that the chances are greater than with other patient groups with curable diseases that the topic of death will surface.

In evocation-type interventions the main thought is not to provoke. Intervention methods, including an open-ended question and reflections, were used as described in subsection reflection vs. intervention page 6. The concept of evocation, which was derived from Carl Rogers’ school of client-centered therapy [72], has been integrated and developed as parts of several interview techniques, including Motivational Interviewing, in which the main emphasis is on non-confronting and non-provocative methods [71]. In this study, the open-ended question used was: “*What changes can you see taking place in the future*?” (See reflection vs. intervention page 6). The complex reflections used varied depending on the context.

Evocation may be described as an invitation to enter into difficult discussions. Evocation to facilitate death talk does thus not necessarily lead to death talk.

The following are examples from clinical interviews where the same evocation statement renders two different outcomes:

Patient A: “I worry about my children”.

Chaplain: “You are worried about will happen to your family when you are no longer there for them.”

Patient: “Yes and I do not know how to discuss this with my children, especially my son. Is it possible that you could bring this to them gently before I talk to them?”

Chaplain: “You may have a short time left to live and you want me to prepare your children for that.”

Patient: “Yes. Can you do that?”

Chaplain: “Yes, I can do that. Do you want to tell me how you think about your situation?”

Patient B: “I worry about my children”.

Chaplain: “You are worried about will happen to your family when you are no longer there for them.”

Patient B: “Well, I have been thinking that maybe it is time for me to step down as chairman in the family company and I want my older daughter to take over. I am worried that my son may feel left out and that may lead to conflicts within the family. I was thinking that maybe someone like yourself could talk to them and help them settle this matter.”

Chaplain: “You want me to talk to them about changed roles in the family business when you are no longer there to run things.”

Patient B: “Well, is that not a part of your work? To help families, I mean, family counseling?”

Chaplain: “I can be there when you tell them. If that is what you want?”

Patient B: “Yes, yes, that would be great. Thank you.”

Chaplain: “Is there anything else you want to discuss?”

Patient B: “Well, the food in this place is terrible. Do you get the same crappy food?”

### Opening statements

Opening statements used by patients at the beginning of the interview were systematically recorded and thematically categorized. The process was deductive. We were looking for indications of whether or not the patients wanted to discuss death. Some of them lead to a discussion of their own impending death (Table 2), but the opening statements showed very different ways of starting the interview, and it was difficult to see at the beginning of the interview, based on opening statements, if the interview was moving towards death talk. The approach to the interviews was based on the ideas of Cicely Saunders concerning “being with” terminal patients [19,29]. For Saunders listening was a big part of her notion, and ‘being with’ patients was achieved through listening. Listening is, therefore, an essential skill when caring for people with terminal disease. It is through listening that we learn what the patient wants. When discussing death it’s important to turn from death as negative to an opportunity for growth. In order to do that it is necessary to take care of the person as a whole, not only his/her physical needs. Therefore, we attempt to learn more about the patients’ real wishes by analyzing opening statements. Also, to make sure that we are not missing any signals from the patient about being open to a discussion about their own imminent death [19,29].

**Table 2:** Opening statements leading to spontaneous death talk during the first interview including 65 women and 34 men

	***Women***		***Men***	
	**30-69 years-old**	**70+ years-old**	**30-69 years-old**	**70+ years-old**
	**n = 33**	**n = 32**	**n = 19**	**n = 15**
Acceptance	6%	9%	0%	0%
	(2/33)	(3/32)		
Concerns about well-being of family or family crisis	33%	9%	26%	0%
	(11/33)	(3/32)	(5/19)	0%
Existential/spiritual/religious/pastoral	58%	81%	58%	87%
	(19/33)	(24/34)	(11/19)	(13/15)
Remorse	3%	0%	16%	13%
	(1/33)		(3/19)	(2/15)

Opening statements were deductively categorized as follows, based on themes, to facilitate comparison of the prevalence of various topics:

*Acceptance*: These comprised statements indicating that patients were at peace with the fact that they were dying e.g. “This has been a good life so I accept death”, or “I look forward to dying”.

*Concerns about well-being of family or family crises*: These comprised statements like “I am worried about the well-being of my family”, or “What will happen to my family?”

*Family member encouraged referral*: This category was assigned if patients opened interviews with statements like “My partner wanted me to talk to you about my situation”.

*Existential*: These comprised opening statements that included metaphysical problems, which are known in other situations but here they are connected to terminal illness, such as “Does life have a purpose?” or “Does God exist?” or “Is death merciful?”

*Spiritual/religious statements*: These included statements like “Will God forgive me all my sins?” or “Does it hurt when the soul leaves the body?”

*Pastoral statements*: These statements are historically rooted in Pastoral Care at a person’s deathbed. These comprised statements like “Can I talk to you about not believing in God?” or “Will you pray for me?”

*Ambivalence*: The difference between ambivalence and denial is blurry in an environment where you have terminal patients. These comprised opening statements indicating that patients thought they were not dying, for example, “I keep fighting” or “I hope I’ll get well soon” or “I’m getting better”.

*Physical concerns and issues involving care*: These included statements in which patients referred to their physical condition, pain, and their care by hospital staff such as “It hurts so much, it’s like it’s never going to end” or “My heart is weak” or “I’m angry with my doctor…he doesn’t care for me”. In all these interviews the patients ended up talking about their own impending death. Some of them said that they felt death coming through the pain and hardship of their physical body.

*Remorse*: These included statements like “In my lifetime I’ve done some bad things” or “I have wasted my life” or “I have abused a lot of people”.

*Unspecified/other*: These opening statements didn’t fit any of the above categories and included statements like “I don’t have much to talk about” or “I don’t want to talk about cancer” or “Are you about my age?”

It is clear that the opening statements didn’t always involve an invitation to talk about death, and, therefore, the interviewer must stay open to such invitations through the entire interview.

### Statistical analysis

When appropriate, the significance of differences between groups was calculated, mainly by using relative risks and determining 95% confidence intervals. P-values were calculated using Fisher exact tests. Power analysis showed that approximately 130 patients (65 women and 65 men) would need to be recruited to detect an approximately 20% gender difference at the p < 0.05 level.

## Results

During the data collection period 195 interviews were conducted. The age distribution of men and women was similar (Table 1). Of the men, 91% (104/114) were receiving terminal cancer care, as were 88% (71/81) of the women.

Table 3 shows that 80% of the women and 30% of the men interviewed had spontaneous death talk with the chaplain within the time frame of 30 minutes. After evocation-type interventions, 59% (67/114) of the men and 91% (74/81) of the women engaged in death talk (Table 3). However, even with evocation, the differences between the genders were still statistically significant (p = 0.001), the initial gender difference in death talk of 50% (30% men versus 80% women) before the first evocation-type intervention having decreased to a difference of 32% (59% versus 91%, respectively) by the end of the first interview.

**Table 3:** Death talk by gender

	**Male**	**Female**	**RR (95% CI)**	**Fisher exact**
	**N = 114**	**N = 81**		**P-value**
Fi*rst interview:*				
Death talk initiated by client within 30 minutes	30%	80%	0.37 (0.27-0.50)	<0.001
	(34/114)	(65/81)		
Death talk after chaplain’s evocation*	59%	91%	0.64 (0.54-0.76)	<0.001
	(67/114)	(74/81)		
*Second interview:* #				
Participating in second interview	36%	29%	1.27 (0.49-4.59)	1.000
	(17/47)	(2/7)		
Chaplain’s evocation leads to death talk*	29%	0%	NR**	1.000
	(5/17)	(0/2)		
No death talk after two interviews	37%	9%	4.26 (2.10-8.98)	<0.001
	(42/114)	(7/81)		

*Either as the result of direct reflections during the interview or in response to the open ended question at the end of the interview.

^#^Of the 47 men and 7 women not engaging in death talk at first interview, 17 men and 2 women booked additional interviews.

^**^Not able to calculate significant levels due to few observations.

Seventeen of the 47 men (36%) and two of the seven women (29%) who did not engage in death talk during their first interview asked for a second interview. Five of these seventeen men (29%) engaged in death talk in response to evocation-type interventions during their second interviews, but neither of the two women did (Table 3). Thus, the total proportion of “no death talk” by the end of the second interview was 37% for men versus 9% for women, reducing the gender difference to 28% (Table 3). Thus, gender differences were still statistically significant (p = 0.001); however, the relative gender difference had decreased.

Categorization of statements used by patients to open discussions about their own impending death is presented in Table 2. Statements most commonly used by both women and men to initiate death talk were existential/spiritual/religious/pastoral statements or concerns about well-being of family and family crisis (Table 2). A gender difference was observed regarding the opening statement categorized as “acceptance”: no men fitted that category and the word “remorse” was uttered by five men and one woman. Concerns about well-being of family or family crisis were common in both younger men and younger women (Table 2).

## Discussion

A clear gender difference regarding the prevalence of death talk was observed. Although evocation significantly reduced this difference, it remained statistically significant throughout the study period.

The importance of “being with” dying patients is emphasized in Saunders’ pioneering work [19-30]. However, because of lack of time and adequate training in having discussions with patients about their impending deaths, health care personnel may find it hard to live up to these expectations. Use of non-provocative evocation-type interventions in conversations with terminal patients in hospital settings to stimulate discussion on their impending death poses a challenge for health care personnel.

### Gender aspects

In the present study, the majority of women spontaneously initiated talk about their own impending death during interviews. In the same situation, far fewer men initiated death talk. However, a large proportion of men responded to simple evocation-type interventions, indicating that they were open to talk about their own death, but needed evocation to do so. These gender differences persisted throughout the study but were significantly reduced by the evocation-type interventions.

#### Expressing emotions

The findings from the present study support previous findings from a population-based study Sweden that men may be reluctant to share emotionally taxing feelings [32]. A recently published study from a nationwide population-based survey of Icelandic widowers reported similar indications of resistance to expressing emotional concerns. The study showed that, in spite of a strong interest in the subject, a majority of widowers identified emotional obstacles to participation in a questionnaire survey on bereavement [73]. Previous findings from a population-based study in Sweden demonstrated a significantly lower prevalence of “emotional isolation” in middle aged and elderly women [74] compared with men [32].

The results of these studies support those of the present study in that women appeared to be more at ease with expressing and discussing emotionally difficult subjects. Although most men may have similar needs to participate in emotional discussions, they seem more likely to need encouragement to do so (Table 3). In the present study, approximately seven in ten men and two in ten women responded to evocation-type interventions to initiate discussions about their own impending death.

### Ethical dilemma

All participants were aware of the fact that they were dying, and had requested interviews with a chaplain. According to generally accepted interpretations of Icelandic law on the rights of patients, health care professionals are not permitted to discuss patients’ prognoses with their families without the patient’s consent unless the patient is unconscious or mentally unable. This limitation poses ethical problems. On the one hand, research has shown that absence of timely insight and communication regarding a patient’s impending death, patient’s significant others may subsequently experience long-term psychological distress [33,35,38-43]. On the other hand, such discussions with patients significant others cannot take place without the consent of the patient. Consequently, timely discussions between a health care professional and patient about patients imminent death are a perquisite for timely communication with the patients significant others. At the same time the patient’s own reluctance to accept death needs to be respected. This ethical dilemma had resulted in a serious dialogue amongst hospital staff at the University Hospital in Iceland.

To address this problem, the systematic evocation method was developed and introduced into clinical practice. The method is known e.g. within Motivational Interviewing [71]. It is a non-confrontative method, which opens up ways to give patients an opportunity to enter into death talk in a safe environment. The patients were thus in a clinical process. The data which was gathered was comparable to patients’ medical records. At the time of the interviews neither the patient nor the chaplain was aware that the content of the field notes would be subject to future analysis and scientific study. The possible effects of the evocation had not previously been studied.

### Communication

Research and training in end-of-life communication is a growing field in health care. The potential benefits of communicating accurate information regarding prognosis are several; both from the perspective of dying patients and of their loved ones. However, despite several reports concluding that patients want full disclosure about their illnesses, many seriously ill persons may not be ready or able to receive prognostic information [75].

The main rule for high quality clinical communication is to always listen actively to how patients respond to open-ended questions and to reflect their answers back to them to offer a way to have a discussion on emotionally difficult issues, like their own impending death. Findings from a clinical study assessing acquisition and retention of evocation skills indicate that, although the theoretical principles behind clinical communication methods like Motivational Interviewing may be relatively simple and easy to understand, it takes considerable time and supervised training to achieve competency in the method [76].

There is no clinical consensus or guidelines for this area of care giving. Few clinicians have documented and published in a scientific/systematic way what happens in such clinical interviews. This lack of scientific documentation is probably due mainly to heavy workloads. People tend to develop their own personal ways of conducting patient interviews and often use systematic documentation. Their experience and knowledge may thus be passed on through clinical supervision, but are rarely published in scientific journals accessible to all.

### Ambivalence

Some patients are ambivalent, and may suppress or even deny the reality of their own imminent death as a way of coping. There are few studies in this area. One study from St. Christopher’s Hospice in London showed that 26% of respondents partially suppressed awareness of impending death, whereas 8% demonstrated obvious denial in the last 8 weeks of life [77,78].

One study on cancer patients in their final few weeks of life found that 17% had “partial awareness”, 9.5% “denying awareness” of both their terminal prognoses and foreshortened life expectancies. Depression was almost three times greater among patients who did not acknowledge their prognoses [79].

### Duration of awareness

A Swedish study that examined widowers’ preparedness before their wives’ deaths from cancer and how it affected their risk of long-term morbidity found that lower preparedness increased emotional numbness, grief resolution, and sleep disorders 4–5 years after the deaths of the participants’ wives [41]. Another Swedish study found that wives’ awareness time concerning their husbands’ impending death from cancer was influenced by information and psychological support from caregivers. Fifteen percent of the wives reported duration of awareness of 24 hours or less. A short awareness time was associated with additional, but avoidable, psychological trauma [35].

### Opening statements

The analysis of opening statements leading to death talk (Table 2) is not to be seen as indications of the prevalence of these concerns in terminal patients. They should only be taken for what they are, namely spontaneous “opening statements” in a clinical pastoral setting.

Although the pastoral context of the interview probably influenced the opening statements identified in the present study, they may have value for other health care professions dealing with similar issues and could provide a first step for the development of training material for Palliative Care personnel. Indeed, these findings are being used by the present team as a partial basis for developing such training material for health care professionals in Iceland. However, development of such material needs to be evidence based and ethically sound.

### Methodological strengths and weaknesses

One strength of the present study is that the patients interviewed comprised all terminal care referrals received by one chaplain (BS) during the course of 3 years (2006–2008). To minimize the risk of errors due to inexperience and different interview techniques, it was decided to use only the most experienced grief counselor of the Palliative Care team for this study. However, this is also a weakness of the study because it may limit the generalizability of the findings.

There are several obvious problems regarding generalizability. Firstly, the findings may only be valid in countries with similar cultural backgrounds. Also, all participants asked to see a hospital chaplain. The role of the chaplain may to a large extent explain the types of opening statements presented in Tables 2 and 3. The hospital chaplain (BS) who conducted the interviews is a middle-aged man who is relatively well known in Icelandic Palliative Care. Iceland being a small country (population of approximately 321,000), this may have affected the content of the clinical interviews and the characteristics of the study population. Although all patients requesting Pastoral Care when BS was on call were included and thus should be representative of end-of-life care patients in general at the relevant clinics, it is possible that some patients may have actively decided to ask for pastoral care when this particular chaplain was on call. For example, it may explain why there were proportionally more men (*n* = 114) than women (*n* = 81) patients among the study participants. Also, the study only included people aged 30–75 years. The older age limit (75 years) was set to minimize inclusion of senile patients, whereas the younger age limit (30 years) was set to avoid having too few individuals in each category (because terminal illness is less common in younger persons).

The present study uses quantitative methods commonly used in medical studies for presentation of the findings. However, the data collection is based on methods developed in Anthropology and thematic content analysis is used to identify subject matters most common in the interviews. Although using methods from different disciplines may sometimes be the most feasible way to assess some aspect of medical practice, this complicates the methodological focus. In general, medical studies using methodological approaches drawing from other disciplines are underrepresented in medical scientific databases [80].

At the Icelandic National Hospital end-of-life discussions are most commonly conducted by a hospital chaplain specialized in grief counseling. We obviously cannot guarantee that no death talk took place between patients and other members of the health care team during the time of the study. However, because they are not usually documented in patients’ case notes, it is impossible to control for such discussions if they take place.

### Future research

The present study is executed within the framework of chaplaincy services. Future studies may need to asses other disciplines like specialized nurses and physicians in Palliative Care. Also the prevalence of death talk and the evocation method needs to be tested more thoroughly in other cultural settings.

## Conclusions

Men in terminal care seem more reluctant than women to enter into discussion regarding their impending death.

Gender differences in terminal care communication may be radically reduced by using simple relatively unpretentious evocation methods, but require considerable clinical training.

Intervention based on non-provocative evocation methods may increase death talk in both genders, the relative increase being higher for men.

## Competing interests

The authors declare that they have no conflicting interests relating to this study.

## Authors’ contributions

BS conducted all patient interviews and recording of verbatim interview field notes, actively participated in the planning of the data analysis, led the writing of the paper, and was responsible for correspondence with the Icelandic data protection and ethical authorities. AH participated in the analysis for Table 3 and helped with the theoretical background and discussions. KA assisted with the statistical analysis, tables and results section. ARH supervised the study and the analysis and writing of the paper. All authors read and approved the final manuscript.

## Pre-publication history

The pre-publication history for this paper can be accessed here:

http://www.biomedcentral.com/1472-684X/13/8/prepub
